# Enhancing the diagnostic potential of electroretinography in Parkinson's disease: A review of protocol and cohort criteria

**DOI:** 10.1177/1877718X251331863

**Published:** 2025-04-29

**Authors:** Victoria Soto Linan, Marc Hébert, Martin Lévesque

**Affiliations:** 1 Department of Psychiatry and Neurosciences, Faculty of Medicine, Université Laval, Québec, QC, Canada; 2 Integrative Neuroscience and Experimental Therapies Axis, CERVO Brain Research Center, Québec, QC, Canada; 3 Department of Ophthalmology and Otorhinolaryngology, Université Laval, Québec, QC, Canada

**Keywords:** Parkinson's disease, electroretinography, biomarkers, diagnostic technique, human, early diagnosis

## Abstract

Electroretinography has emerged as a promising tool for identifying retinal functional anomalies in major psychiatric and neurodevelopmental disorders, such as schizophrenia, major depressive disorder, bipolar disorder, and autism spectrum disorder, positioning it as a potential biomarker of monoaminergic dysfunction. However, despite its potential, electroretinography studies in Parkinson's disease (PD) over the past decades have been inconsistent, largely due to variations in research methodologies. These limitations diminish its potential and hinder the association between retinal electrophysiological responses and PD neuropathology. To address this challenge, this review examines the most relevant sources of data variability and reduced reproducibility in electroretinography studies aimed at detecting a retinal functional signature characteristic of PD. We propose the consolidation of four key protocol factors and five cohort criteria to enhance the diagnostic accuracy of electroretinography in PD biomarker research. As electroretinography protocols are adapted from their clinical origins for research purposes, we argue that careful attention must be given to electrode type and placement, as well as to factors like age, sex, disease duration and severity, medication intake, psychiatric conditions, and comorbidities in cohort selection to ensure reproducible results. Suggesting that past inconsistencies in these areas may explain the variability in reported results and contribute to the lack of consensus on which electroretinography parameters comprise a disease signature in PD, we ultimately offer recommendations to improve the utility of electroretinography techniques as early biomarkers for PD.

## Plain language summary

Electroretinography is a non-invasive test that measures how well the retina—the part of the eye that senses light—works. This technique has shown promise for detecting retinal irregularities in conditions like schizophrenia, depression, bipolar disorder, and autism, and it might help detect early signs of Parkinson's disease. However, past studies have had inconsistent results, making it hard to confirm the effectiveness of electroretinography in diagnosing Parkinson's disease. We reviewed the sources for these variations, focusing on how studies were conducted and how participants were chosen. Our goal is to suggest ways to improve electroretinography testing for Parkinson's disease research. We recommend prioritizing key aspects like the type and placement of electrodes (small devices placed on the eye to measure electrical signals) and paying close attention to factors like the person's age, sex, disease duration, and medications. By addressing these elements, we believe electroretinography could become a more reliable tool for identifying early signs of Parkinson's disease. Researchers should concentrate on standardizing these testing methods to ensure more consistent results and increase the feasibility of early diagnosis.

## Parkinson's disease, the retina, and electroretinography anomalies

Parkinson's disease (PD) is a progressive motor disorder caused by the degeneration of dopaminergic neurons in the substantia nigra pars compacta region of the midbrain. It is primarily characterized by motor dysfunctions such as resting tremor, rigidity, and bradykinesia, which manifest after significant dopaminergic neuronal loss.^1,2^ However, the problematic remains that a diagnosis of PD is mainly reached after the appearance of motor symptoms and supportive criteria, with the diagnosis being confirmed postmortem.^3^ At this point, there is already substantial and irreversible neurodegeneration. Existing treatments may only ameliorate symptoms, but there is a limitation on their efficacy with such a late diagnostic time point. Nevertheless, patients with PD often complain of visual disturbances, such as blurry vision, in earlier stages of the disease. With progression, a large proportion of Parkinsonian patients also suffer from visual system deficits that manifest as significant errors in chromatic discrimination and reduced contrast sensitivity.^4^ Although motor symptoms of PD can be attributed to the prominent dopaminergic dysfunction seen in the midbrain, it seems that pathological non-motor disturbances of the retinal tissue arise earlier via a less clearly understood mechanism. As these visual disturbances present themselves in the early stages of pathology, retinal anomalies have been proposed as an early biomarker of disease in PD.^5,6^

**Figure 1.**
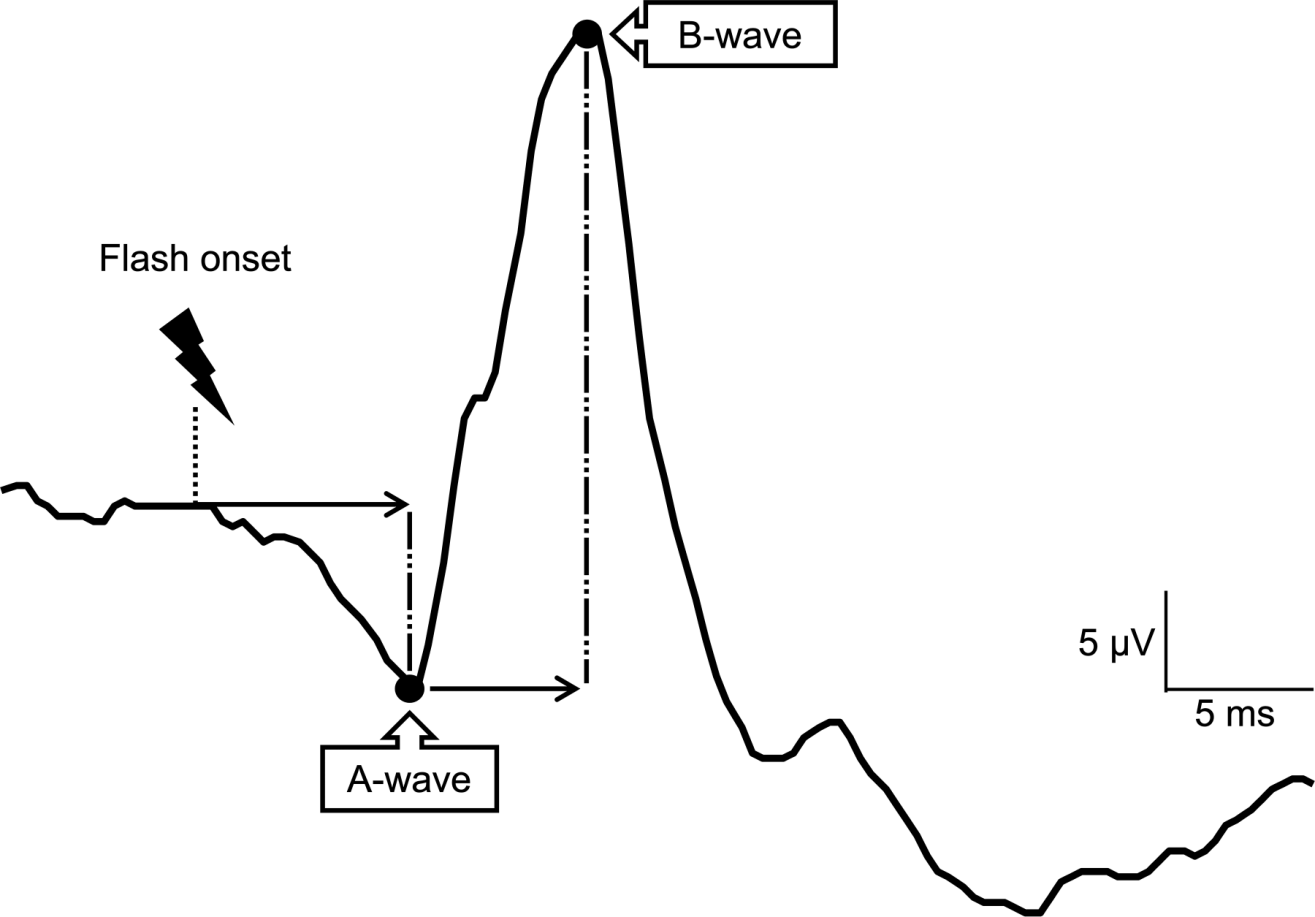
Representative human ERG wave trace. This photopic trace demonstrates the two main ERG wave components commonly used in ERG studies, i.e., a-wave and b-wave. Following flash-light stimulus, the former is negatively deflected, and the latter follows with a positive deflection. Each ERG component is characterized via latency (ms) or the speed of the response, and the amplitude (µV) or the strength of the response.

During the embryonic development, the retina and optic nerve extend from the diencephalon and are therefore considered part of the central nervous system. However, unlike other parts of the central nervous system, it remains relatively accessible for functional assessment in live subjects. The retina is also a well-studied tissue so that alterations to the healthy structure are easily detectable. Moreover, due to its well-defined neuronal compartment, the retina is a prime candidate for studying mechanisms of pathology and reactions to pharmacological agents in their intact form.^7^ These properties have been leveraged using the non-invasive technique known as electroretinography (ERG), which is used in both clinical and research settings to assess retinal function.^8–10^ This technique directly measures the overall change in the electrical potential of the retina in response to light stimulation.^11^ Physiologically, the light-induced retinal field potential is generated by circulating ion currents of rods^12^ and cone photoreceptors^13^ and can be sectioned into ERG components called waves. In a single ERG reading, light stimulus first results in the hyperpolarization of the outer segments of photoreceptors in the outer retina, creating the negatively deflected a-wave component.^14^ Next, the depolarization of bipolar cells and Müller cells in the inner retina generates the positively deflected b-wave component.^15^ Additionally, oscillatory potentials are found along the ascending part of the b-wave, most likely generated by the interaction between amacrine and bipolar cells.^10,16,17^ Recording such traces in both scotopic (dark-adapted) and photopic (light-adapted) conditions further assesses the rod or mixed rod-cone and cone system contribution to each of the ERG components, respectively.^18^ Ultimately, analysis of an ERG trace is accomplished via amplitude and latency characterization of each ERG component (Figure 1).

Of particular interest to PD is understanding the connection between the electrophysiological changes denoted by each ERG component, the retinal cellular populations behind each response, and dopaminergic dysfunction. In the human retina, dopaminergic neurons reside among amacrine cells. Dopamine containing neurons, on the other hand, are present as inter-amacrine cells that ramify in the inner plexiform layer, usually near its junction with the inner nuclear layer. They also exist as inter-plexiform cells present in the inner and outer interplexiform layers, which receive inputs from amacrine cells; however, they end in bipolar and horizontal cells. Specific ganglion cells in the mammalian retina may also use dopamine as their neurotransmitter to communicate with central visual areas.^19^ Further supporting dopamine's role as a neurotransmitter in the retina, potassium-driven depolarization of the human retina releases endogenous dopamine stores through a calcium-sensitive mechanism usually present in phototransduction.^20^ Remarkably, in the retinas of PD patients, pathological visual deficits can lead to abnormal electrophysiological responses such as multifocal ERG decreased electrical activity in the fovea^21^ as well as thinning of the retinal nerve fiber layer.^21–24^ Such anomalies seem to be caused at least partly by the dopaminergic deficiency of PD patients driven by reduced expression of tyrosine hydroxylase (the rate-limiting enzyme in dopamine synthesis). It has also been suggested that central dopaminergic deficiency in PD may be indirectly linked to the retinal dysfunction observed in patients. This is evident from studies that have found that dopamine precursor administration can ameliorate compromised visual-evoked potential latencies that are otherwise delayed in PD patients. Dopamine precursor administration also has a similar effect on healthy subjects with administered dopamine blockers.^25^ Studies using a dopamine receptor blocker such as thioridazine on human subjects, showed a decrease in ERG retinal rod sensitivity, prolonging the scotopic b-wave latency and reducing the corresponding b-wave amplitude.^26^ Similarly, a recent study utilizing a dopamine transporter knockout mouse model yielding a fivefold increase in brain extracellular dopamine was accompanied by a decrease in ERG retinal rod sensitivity. Surprisingly, in this latter study, despite dopamine fluctuations in the brain, there were no changes in retinal levels, unlike what has been reported in previous postmortem studies of PD patients’ retinas.^27^ This suggests that central dopamine neurotransmission can affect ERG responses and that anomalies reported might serve as biomarkers of central monoaminergic dysfunction.^17^ In fact, in PD patients, past ERG studies have shown a similar reduction in cone and rod b-wave amplitude, supporting a representation of the central dopaminergic pathology creating alterations at the retinal level.^28^ Therefore, it can be concluded that dopamine is closely involved in modulating retinal signal transmission and output. As this is most pertinent in PD, the ERG becomes a valuable diagnostic technique by measuring such downstream retinal output fluctuations.

Nevertheless, despite our current understanding, not all studies seeking to use the ERG as a biomarker of PD have yielded similar results. Older studies of ERG report reductions in scotopic b-wave amplitude, photopic a-wave and b-wave amplitudes, and prolonged latency of the photopic b-wave.^29^ Others describe a reduction in scotopic a-wave amplitude, scotopic oscillatory potential amplitude, photopic b-wave amplitude, and the prolongation of mean peak times of the scotopic oscillatory potentials.^30^ More recent ERG studies suggest that PD patients have lower photopic oscillatory potentials, photopic b-wave, and photopic negative response amplitude in the absence of structural damage.^31^ Yet, another ERG study, which also encompassed visual evoked potentials and pattern-ERGs, reports a significant reduction in the scotopic b-wave and photopic a and b-wave amplitudes, accompanied by variably reduced second photopic oscillatory potential.^28^ Lastly, there have been others for which PD patients and healthy subjects exhibited no significant difference concerning both a-wave and b-wave latency and amplitudes in the scotopic rod and blue flash ERG, photopic cone ERG, and b-wave maximal combined rod/cone response.^32^ However, it can be argued that the lack of consensus among these studies does not necessarily deprive the biomarker potential of the ERG. As previously proposed, a biomarker is defined as an objective measure of normal biological and pathogenic processes. For a biomarker to be ideal, it must be linked to fundamental features of PD neuropathology and be validated in confirmed cases of PD. Most importantly, at its core, it must detect PD early with high sensitivity and specificity, be non-invasive, simple to perform, reproducible, and inexpensive.^33^

**Figure 2.**
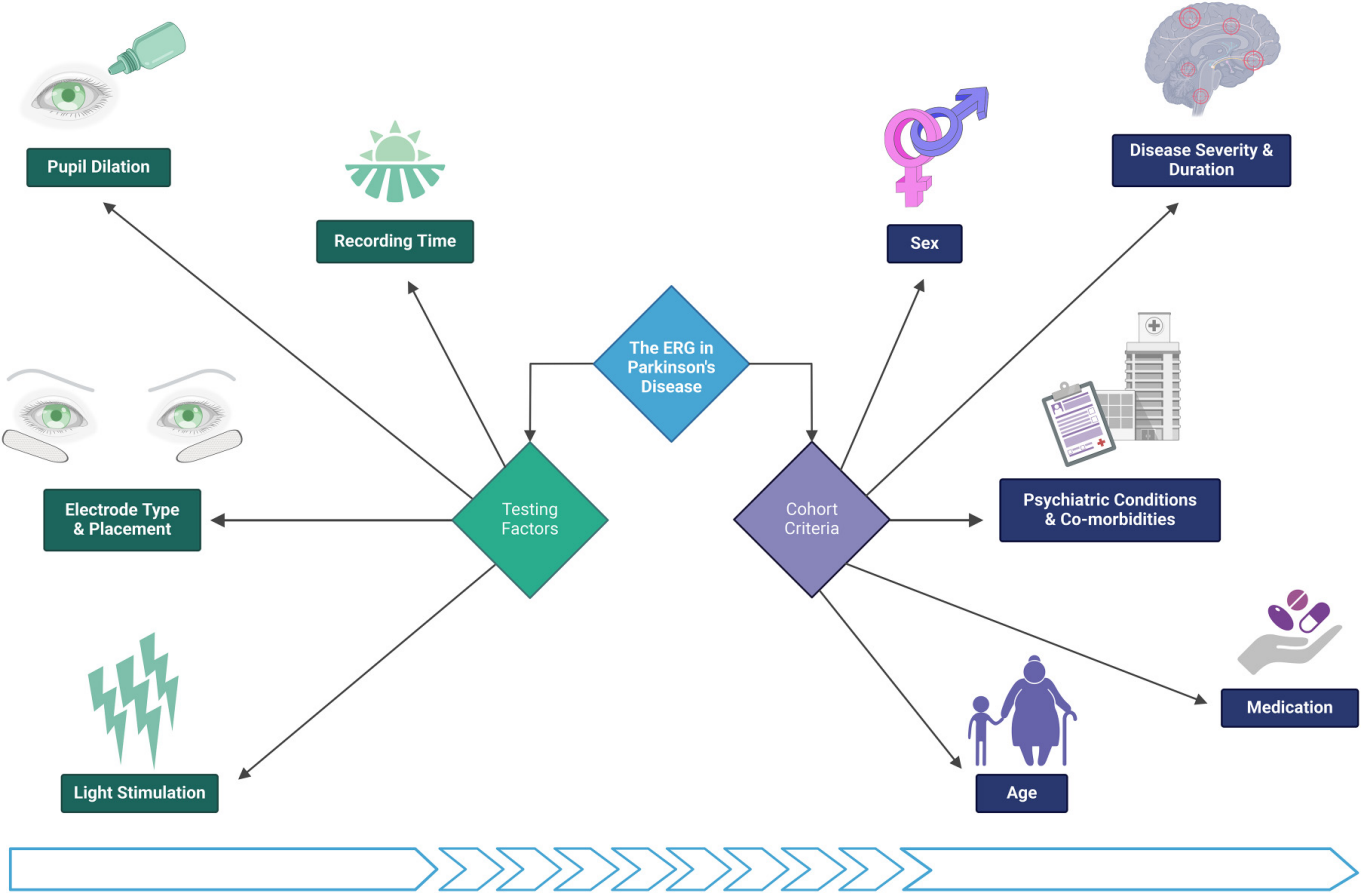
Schematic of ERG factors and cohort criteria of importance in ERG studies of Parkinson's disease. The outlined key details should either be tightly regulated or accounted for when using ERG to identify early PD anomalies. *Created in BioRender. Soto Linan, V. (2025) BioRender.com/g99b685.*^35^

While the ERG meets several of these criteria as a biomarker, our current limitation is that if we are to define an ERG signature that is characteristic of PD anomalies, research studies should be able to report a more uniform pattern across all ERG components. Therefore, we seek to review previous ERG studies by identifying specific factors to consider in diminishing variability and refining the reproducibility of the data, ultimately improving the potential for the ERG to be used as an early diagnostic biomarker^34^ in PD (Figure 2). Should we be able to establish a more standardized protocol and criteria, as has been done for other disorders, this relatively non-invasive tool (depending on the electrode type employed) has the potential to detect existing anomalies in retinal tissue disturbances resulting from ensuing pathology. ERG, in partnership with additional biomarkers, might allow for PD to be diagnosed at its pre-motor symptomatic stage. This also raises the possibility of its use beyond diagnosis, including as a predictive, susceptibility, and monitoring biomarker.^34^

## ERG protocol components

Among other types of electroretinography tests measuring retinal function and potentials (such as multifocal-ERGs, pattern-ERGs, and visual evoked potentials), the International Society for Clinical Electrophysiology of Vision (ISCEV) has established standards for full-field ERG. These protocols are updated every four years and outline minimal clinical standards, including light stimulation, electrode types, pupil dilation, and several others.^36^ However, it is essential to note that these standards are directed for ophthalmological application and standardized clinical establishment and report of eye diseases. In research, they are therefore not mandatory and are instead used to promote uniform ERG methods and protocols.^37^ Moreover, these do not exclude tests that are not covered by the ISCEV. As a result, electrophysiologists are encouraged to extend test protocols as required when relevant to maximize the ERG's diagnostic value for patients and human-based research.^36^ With this in mind, we highlight four main factors in ERG practice that should be carefully considered in PD research, not only because of their pre-established clinical importance to ERG but also because of how particularly sensitive they are in PD.

### Electrode type and placement

The first factor in an ERG test that is of importance is choosing the electrode type and placement that enhances readings and reduces the signal-to-noise ratio the most. It should be noted that there is no ideal electrode in ERG studies, and thus, several aspects must be weighed in the pros and cons of the type chosen. This explains why, in the past, electrode choice and placement have varied in electrophysiological studies of PD cases. Previously, irrespective of the model used, corneal contact lens electrodes such as Lovac contact lens, ERG-Jet lens electrodes, or the Burian-Allen lens electrodes yielded ERGs of large amplitude and high signal-to-noise ratio with minimal intrasubject and intersubject variability, making them the most potent electrodes. This is attributed to the electrode's optimal electrical contact with the eye and the use of a blepharostat, which keeps the eye open during the procedure, maximizing the stimulating conditions. However, even these ideal probes are accompanied by limitations such as the inability to fit small palpebral fissures, the need for topical anesthesia and cooperation from the subjects, anxiety-induced events in participants, the risk of corneal abrasions, the rising cost of such an electrode, as well as deterioration of the optical quality of the eye that hinders pattern-ERG studies. That is why less intrusive electrodes were developed, such as the Dawson, Trick, and Litzkow (DTL) that provide a higher level of comfort for users and also allow for longer recording periods with no need to anaesthetize the eye.^38–40^ While the signal amplitude may be lower with the DTL compared to contact lens electrodes, by averaging more waveforms, very high-quality and reproducible readings can be attained.^41,42^ Yet, another type of even less invasive electrodes—skin electrodes placed below the lower eyelid—exists, but require further averaging to be suitable for detecting attenuated pathological ERGs^36^ due to the decrease signal that can be captured through the skin and the higher noise ratio.

The electrodes chosen by past teams performing electroretinography testing on PD participants ranged from jet electrodes,^31^ contact lens,^29,30,43^ and gold cup^30^ electrodes to corneal,^28^ DTL fiber,^44,45^ and adhesive skin^46,47^ electrodes. Visual evoked potential studies relied on surface scalp electrodes,^28,43–46,48^ whereas remaining pattern-ERG ones either focused on gold foil,^28^ corneal,^44^ DTL,^48,49^ or skin electrodes.^46^ Lastly, focal-ERG, flash, and pattern-visual evoked potential combination studies have incorporated skin and surface scalp electrodes.^46^ This means that data variability was likely first introduced with the choice of electrode within similar testing procedures. Even among studies using the same electrode fibers, the placement of such has not always been the same and is likely the second source of induced data variability. For example, at times, DTL electrodes were referenced and grounded to the ipsilateral and contralateral temples, respectively.^48,49^ At other times they were referenced to the outer canthus and grounded to the frontopolar plazierte.^44^ DTL placement can also be situated in the conjunctival sac or just below the iris on the eye. While the latter positioning being closer to the cornea can double the amplitude of the signal that is captured, it can also be more prone to displacement during recording, thus affecting reproducibility. Similarly, contact lens electrodes differed in grounding or reference to the earlobe^30^ or forehead.^29,43^

Although a biomarker's primary role is to accurately and reliably reflect a biological process or disease state, its clinical utility is further strengthened by non-invasiveness and ease of administration. Thus, in aiming to use the ERG as a biomarker and diagnostic aid, PD researchers should choose an electrode type and placement with high reproducibility and measuring power that also caters to the needs of these patients. For this, we propose two candidates, i.e., DTL and skin electrodes. DTL electrodes consist of a nylon fiber impregnated with silver that is best placed deep in the inferior conjunctival sac for comfort and secured at the inner (nasal) canthi. While wave amplitudes are around 50% lower than when performed touching the cornea,^39^ this positioning is also less prone to test-retest variability due to displacement during the recording, allowing for a higher likelihood of differentiating between healthy and clinical populations.^50^ Contamination with photomyoclonic artifacts arising from the absence of a blephorostat can be minimized with the use of a reference electrode on the outer (temporal) canthi and a ground electrode on the forehead.^39^ To achieve this level of reproducibility, first, the electrode's position must be consistent since the absolute amplitude of the ERG recorded varies relative to the electrode's position from the center of the cornea.^51^ Second, the fiber must be placed loosely in the conjunctival sac as this minimizes the risk of displacement and avoids sudden changes in the absolute amplitude of responses.^41^ Using the DTL electrode and all of the criteria mentioned above allowed a study on around 100 consecutive clinical patients from all age groups ranging from 6 months to 70 years of age to perform ERG successfully.^39^ For research teams that have clinical resources and that work with prodromal, de novo, or medicated patients, the disposable DTL fibers offer a relatively low level of invasive placement suitable to the clinical stage of the disease. On the other hand, an arguably more ideal electrode for PD patients may be found in skin electrodes. These minimize the amount of training needed from experimenters for placement and maximize comfort for the patient, both factors that must be prioritized considering the symptomatic condition of the disease often found in studies focusing on advanced stages or non-medicated cohorts. While it has been argued that skin electrodes are less suitable for detecting pathological ERG signals,^36^ it is important to note that irrespective of the method of placement used, if additional care is taken to average signals and ascertain proper electrical contact with the skin, the resulting trace should be highly reproducible. In fact, evidence suggests that skin electrodes can offer a viable alternative to their more invasive competitors.^52,53^

### Pupil dilation

The second factor that is part of the ISCEV standards is pupil dilation, whereby in clinical practice, it is ideally dilated, monitored before and after an ERG test, and should be maximal for testing.^36^ As per the retina's topographical arrangement and connection, cones at the very center of the foveal area are connected to a single cone bipolar cell, while at the peripheral retina, many cones connect to a single bipolar cell. That is to say that when not dilated, the light stimulus does not fully reach peripheric cone photoreceptors, thus not ensuring optimal stimulation of the entire retina during testing. Regarding the electrophysiological aspect, lack of dilation reduces the cone-stimulated receptive field. Still, the physiological constraint impacting the amplitude and the latency may be partially corrected by increasing flash intensity and photopic background luminance by 0.5 log units, as demonstrated by Gagné et al., 2010.^54^ Although it can be anticipated that the photopic ERG response would still be slightly lower (14%) in a nondilated eye than in a pharmacologically dilated eye, the pupil size measure could be used as a covariate. In scotopic conditions, the natural dilation of the pupil in the dark, regardless of pharmacological dilation, results in only a minor decrease of 7% in the b-wave maximal amplitude of nondilated eyes.^54^

Electrophysiological studies in PD thus far have used various drugs to ensure pupil dilation. Some rely on 1% Tropicamidum,^28,30,31^ 2.5% neosynephrine,^29^ or cyclopentolate hydrochloride BP (1% w/v).^46^ Others choose to record from nondilated eyes while documenting pupil size.^48,49,55^ Nevertheless, in the research field, it is important to note that implementing such clinical measures would not only present a higher risk and discomfort for participants, but would also require a greater cost from the lab, i.e., participants visiting ophthalmologists prior to each ERG test, clinician-mediated pupil dilation, health care personnel, etc.^56^ Even if implemented, induced dilation has a high interindividual variability.^57^ As with most clinic-based exams, one has also to consider the pupil's state factoring in age. In elderly patients, the pupil dilates significantly less than in younger ones (even when pharmacologically dilated), which may reduce the flash ERG amplitude in both scotopic and photopic tests.^58^ Therefore, when testing PD patients, the term “maximal dilation” of pupils that is desired for ERG readings must be outlined within a research environment, experimental feasibility, patient demographic, and most importantly, closely following biomarker criteria. Since the traits of the cohort cannot be neglected, we propose that unlike initial studies, researchers stray away from pharmaceutical dilation. The field has sufficiently expanded since to support an alternate course of action, i.e., to prioritize tracking the pupil size as a covariate for data analysis^59,60^ and adjusting the light stimulation delivered.^54^ These measures would allow for the ERG to progress as a biomarker while maintaining its non-invasive, inexpensive, and reproducible nature.

### Light stimulation

The third factor to highlight cannot be universally defined due to its intentional malleability in light stimulation steps of a recording session. In the ISCEV standard full-field ERG evaluation, rod and cone function is primarily assessed with five steps: the scotopic pure rod ERG, and combined rod-cone ERG, oscillatory potentials, photopic cone ERG, as well as the 30 Hertz flicker ERG and oscillatory potentials. Briefly, the usual sequence in the presence of corneal lens electrodes starts with responses to low levels of white or blue light in properly dark-adapted subjects to evaluate rod photoreceptor function. The maximal or combined response from both the rod and cone photoreceptors is then elicited with a brighter standard flash of white light. Following light adaptation, cone responses to white flashes are recorded in the presence of rod saturating background illumination. Finally, flicker ERGs recorded at approximately 30 Hertz are used to further ensure measured responses arise from cones only as rod photoreceptors cannot recover rapidly enough to respond to the rapid flashes.^36,61^ Since DTL electrodes do not run the risk of corneal abrasion with extended wear,^62^ the usual sequence otherwise begins with the light-adapted steps and ends with the dark-adapted ones instead. Extended protocols for light-adapted and dark-adapted full-field ERG containing more general guidelines have also been published by the ISCEV.^63^ Although the ERG has not been extensively used within the field of PD research, it is just as important for examiners seeking to establish this technique as a biomarker to be aware of the ISCEV stimulus criteria, for they are designed to follow the stimulation range necessary to reach the activation level of the retinal components tested.^36^ These standard steps pertain to a clinical setting (where pupil dilation is otherwise used) and are summarized in Table 1.^36,64^ As previously discussed, should pupil dilation be avoided and instead accounted for during testing, these standardized steps in non-dilated eyes should be increased by 0.5 log units in both the flash stimulus and the photopic background luminance.^54^

**Table 1.** Summary of light stimulation properties per ISCEV standards.

ISCEV adaptations″	Stimulated retinal components	Detectable ERG parameters	Stimulus Strength (acceptable range)
DA 0.01 ERG	Rod-driven response of ON bipolar cells	Only a b-wave with no detectable a-wave	0.01 (0.0063–0.016) photopic cd·s/m^2^; 0.025 (0.017–0.036) scotopic cd·s/m^2^
DA 3 ERG	Combined rod-dominated responses from photoreceptors and bipolar cells of both the rod and cone systems	Both an a-wave and b-wave (peaking at about 15 and 21 ms)	3.0 (2.7–3.3) photopic cd·s/m^2^; 7.5 (6.1–9.2) scotopic cd·s/m^2^
DA 10 ERG	Combined response with enhanced a-wave reflecting photoreceptor function	Larger a-wave, sharper peak time, clearer b-wave reductions, and enhanced oscillatory potential amplitudes	10 (7.9–13) photopic cd·s/m^2^; 25 (18–34) scotopic cd·s/m^2^
DA 3 or 10 OPs	Responses primarily from amacrine cells	Oscillatory potentials	Photopic and scotopic cd·s/m^2^ of dark-adapted 3 or 10 ERG
LA 3 ERG	Responses of the cone system	a-wave from cone photoreceptors and OFF-bipolar cells; b-wave from ON and OFF-bipolar cells	3.0 (2.7–3.3) photopic cd·s/m^2^
LA 30 Hz ERG	Sensitive cone-pathway-driven response; rods do not respond at this rate	Latency and amplitude of waves	3.0 (2.7–3.3) photopic cd·s/m^2^

″ Serving as a backbone for the tailoring of research protocols, these recommended testing steps in the full-field ERG highlight important parameters and integrate the retinal players targeted in each test. Also included are calibration criteria and considerations as it pertains to each step. Dark-adapted: DA; Light-adapted: LA; Oscillatory potentials: OPs; Hz: Hertz; ERG: Electroretinography; cd·s/m^2^: Luminance in candela second per square meter.

To be more accurate, what the ISCEV provides is a framework used in a clinical setting for the detection of eye diseases that is simultaneously open to adjustment or optimization for research purposes. Therefore, each study should first determine how their objectives will require tailoring of pre-established protocols and how those changes are justified by their research problematic. Should the ISCEV guidelines need to be adjusted, of repeated vital importance are parameters such as the range of flash strengths, flash duration, and background intensity. First, for the light-adapted ERG, the stimulation system must be capable of delivering white flashes across a range from subthreshold (in the absence of dysfunction, this is at 0.1 cd·s/m^2^) to saturating flash strengths. For the photopic cone b-wave component, this is usually achieved between 10 cd·s/m^2^ and 50 cd·s/m^2^, based on our experience with non-dilated eyes. As previously reported, the luminance range serves to define the maximal velocity of the b-wave, which is isn’t obtained at a singular luminance among subjects.^65^ Second, flash duration should always account for the retinal photoreceptors’ integration time (approximately 5 ms), and the flash interval should align with the photopic and scotopic condition and strength of the stimulus.^36^ Lastly, light adaptation for the light-adapted ERG should use a rod-suppressing background to ensure cone system selectivity during testing. The luminance of the background influences the stimulus-response function, where stronger backgrounds require more potent flash stimuli to elicit similar ERG amplitudes, enhance the peak amplitude, and lower the saturated plateau of the b-wave photopic hill. Stimulus-response series recorded with different backgrounds are therefore not comparable.^63^ On the other hand, for the dark-adapted ERG, flash stimuli should range from approximately 3.5 to 4.0 log units since the typical range would be approximately −3.5 to 0.5 log cd·s/m^2^ in the absence of retinal dysfunction. This range would start with the lowest flash strength that will generate a reliable dark-adapted ERG b-wave and would end with the stimulus required to elicit a maximal b-wave.^66^

It should also be noted for consideration that in the recording process of ERG tests, a fixation point should be incorporated into the stimulus dome, and proper instruction should be given so that patients direct their gaze at such point. A stable gaze is essential so that it minimizes eye movements that may alter the position of the electrode and produce electrical artifacts or allow blockage of light by the electrode or eyelid. Fixation points should not interfere with dark adaptation and should be visible in light-adapted conditions.^36^ Besides, informing patients of the central fixation and that each flash would follow a specific time interval would allow them to know when it is ideal or not to blink, optimizing readings.^39^ Additionally, under the most recent ISCEV protocols, recording conditions should abide by 20 minutes (min) of dark adaptation before recording dark-adapted ERGs, and 10 min of light adaptation before recording light-adapted ERGs. Provided the adaptation requirements are met, the examiner may begin with the dark-adapted or the light-adapted ERG.^36^ Lastly, the device used to elicit such stimulations is also worth revisiting. Usually, a stimulus dome is used to ensure proper stimulation of both eyes at the same time. However, an emerging alternative that could facilitate routine testing is the implementation of ERG protocols with a hand-held portable device, such as the RETeval (LKC Technologies, MD, USA). Although this device can test only one eye at a time, its ease of use and low cost could democratize and potentiate the use of the ERG as a biomarker, in which testing both eyes is not a necessity.^67^

As stated previously, the ISCEV structures reference protocols, meant to normalize results in the clinical field. The sensitivity of these protocols and the means required to achieve such results are not readily applicable in the research setting and do not comply with biomarker criteria. That is why, researchers should be aware of what each ERG component measures, the sequence in which these measurements are elicited, and how the recommended stimuli and adaptation periods ensure the activation threshold of retinal cells. Discrepancies in these central points could lead to results that reflect improperly stimulated retinal components should the stimulations applied not be in the same range. As a result, studies would not be able to report comparable data. If we focus on past full-field ERG studies performed on PD patients, while protocols have kept the dark-adaptation period to around 30 min, for light-adaptation periods, protocols have ranged from using 5 min of blue light background (2.2 log troland)^28^ to 2 min of a 6-1fL yellow background created with a Wratten26 filter.^29^ Additionally, these studies have also differed in their flash stimulation parameters during the tests. As a result, although the conclusions reported in several PD studies do not necessarily entirely contradict each other, not all studies consistently report the same parameter changes. We, therefore, propose that teams exercise caution when tailoring their experimental design from the ISCEV frameworks or published experimental protocols by ensuring flash strengths, flash duration, background intensity, and adaptation periods stem from the most up-to-date guidelines, remain consistent, and respect the saturation thresholds of each ERG wave component. Once those are determined, the steps of each test, and thus the length of the testing period, can be reduced to focus on the most pertinent ERG parameters. Without sacrificing reproducibility or reliability, making the ERG simpler to perform would ultimately facilitate rapid testing within patient cohorts and render the ERG a potent biomarker.

### Recording time

The fourth and last important ERG factor to consider regards recording time. As has been previously published, diurnal changes are known to affect the ERG responses and thus must be limited. A study found that a diurnal rhythm exists in full-field rod ERGs elicited from the entrained eyes of healthy human subjects. A decrease in amplitude at 9:30 a.m., 2 hours (h) after light onset, could be detected not only in the b-wave but also in the a-wave components, indicating that this diurnal variation occurs in responses from rod photoreceptors. The presence of a diurnal variation in the rod ERGs of the entrained eyes but not the untrained fellow eyes suggests that this circadian phenomenon is regulated within the eye.^68^ The time course of this rhythm is compatible with anatomic studies that have shown a burst of rod outer segment disc shedding in light-entrained animals soon after light onset in the frog,^69^ rat,^70^ and monkey retina.^71^ In the field, previous studies found that morning and evening variations caused an increase in latency and maximal velocity of the rod b-wave amplitude by 8% and 6% in humans, respectively. However, these changes were considered too small to significantly impact the assessment of retinal function albeit morning recordings were obtained more than 2 h after light onset.^72^ Similarly, while some have argued for the use of the ERG in studying circadian rhythms,^73^ the lack of consistency in the identified retinal rhythms^74^ seem to point towards a persistent limitation. In PD research, past ERG studies have not placed particular emphasis on recording time criteria. However, in the establishment of the ERG as a biomarker, we propose that recording time remain consistent. Therefore, regardless of the light-adaptation or dark-adaptation periods already respected in an ERG protocol, the time in which the recordings are registered must also be considered so that ERG readings intended to reflect PD anomalies are not clouded by diurnal changes or inconsistent retinal rhythms instead.

Altogether, the four ERG-impacting factors discussed thus far need to be carefully respected to ensure that results remain reproducible throughout studies. In the context of PD research, this approach would minimize outside interference to a negligible level, ensuring that the validity of the data remains unaffected. First, the electrode type must be carefully chosen to cater to PD patients. Its location, grounding, and reference electrodes must also be complementary. We recommend using skin or DTL electrodes for their ability, comfort, and reliability, although we cannot exclude other electrodes so long as the same criteria are met. Second, we conclude that over pharmaceutical pupil dilation, more emphasis should be placed on testing of non-dilated eyes. One plausible method can be found via measuring the pupil size and using such measurement as a covariate, especially since pharmacological pupil dilation can still yield differential pupil size between individuals. Third, given alterations in flash stimulus are not discouraged under the ISCEV criteria, which are otherwise intended for clinical use and specificity, we recommend that researchers carefully judge the efficacy and limitations (e.g., pupil dilation) of the strength of the stimulus used to elicit the ERG components they intend to isolate. Fourth and lastly, we recommend that more notice be paid to the time of recordings, specifically avoiding the first 3 h after light onset or wake time to prevent retinal rhythms from impacting an otherwise optimal ERG protocol. We speculate that the differences seen in these four parameters primarily account for incomplete results reported in past studies that allow us to detect PD anomalies under various circumstances, but with no uniformity that can be used for unique distinction and thus, biomarker building.

## ERG cohort participant criteria

With an optimized ERG protocol, the next step is to consider enhanced inclusion or exclusion criteria for the tested cohorts. In human PD studies, the exclusion criteria of the participants are often very well calculated, greatly benefiting from a specialist's input, i.e., neurologist. One aims to reduce the amount of outside interference in the data recorded as much as possible even if these change with the characteristics of a specific experimental group. Given the nature of the ERG, past studies in PD, which have been mentioned thus far, have taken this into account by ensuring an acceptable corrected visual acuity and no eye pathology. However, these should be the base standard of inclusion criteria. To go a step further, we propose that ERG studies for PD research should also consider age, sex, disease severity and duration, medication intake, psychiatric conditions, and co-morbidities.

### Aging factor

As is known, PD affects 1–2 per 1000 of the population at any time, where the onset of the disease is usually at the age of 65 to 70 years. Incidences before the age of 40 are seen in less than 5% of cases in population-based cohorts.^3^ Therefore, most PD cohorts include subjects of advanced age. However, in rod and cone responses recorded by ERG, it is essential to note where the usual line of age deterioration is drawn. In healthy individuals, amplitudes show a gradual slow decline with age up to 55 years and a rapid decline after that. Best-fit exponential functions show that the age at which amplitude drops to one-half that of the young adult range (ages 15 to 24 years) is 69 years for the rod response and 70 years for the cone response. There is also a significant decline in maximum amplitude with age and a slight reduction in sensitivity consistent with the increased b-wave implicit time of the rod response.^37^ This could be attributed to increased load and, as a consequence, loss of the retinal pigment epithelium in the macula, ultimately leading to the accumulation of lipofuscin and photoreceptor death.^75^ While photoreceptors and retinal pigment epithelium cells show parallel changes with aging that are more pronounced in the peripheral retina than in the fovea, it is essential to note that aging seems to affect rod photoreceptors and cells in the ganglion cell layer more than cones.^76^

Researchers forming cohorts that comprise a more extensive age range or an age range situated within the age deterioration threshold in healthy individuals should consider that their readings might be affected by a natural aging factor. In past ERG studies of PD cohorts, this factor seems to have been relatively well-regulated. The average ages of PD participants have ranged from 60.9 ± 7.7 years,^30^ 63.0 ± 9.9 years,^28^ 58.5 ± 11.1,^31^ and 55.7 ± 7 years in PD unmedicated groups and 60.3 ± 5.7 years in PD medicated groups.^29^ Additionally, the control groups for each study have been closely age matched. As previously stated, most ERG studies have found a reduced a-wave^8^ and b-wave amplitude for scotopic conditions^28,29^ and photopic conditions in PD patients.^30,31,47^ Other types of electrophysiological studies have also shown a reduced amplitude in their readings for PD cohorts.^44–46,49,55^ Given that aging also has a similar effect, it is critical to note the influence of age that might be present among the PD anomalies observed. We therefore propose that tight regulation of age range within experimental cohorts continues to be upheld. However, we simultaneously encourage more extensive ERG testing to find additional affected components that may be age-independent, while investigating how such ERG components evolve with age and consequently, disease progression.

### Sex differences

A secondary factor that should not be overlooked is the suggested effect of hormonal input^77^ and sex differences on ERG recordings, whereby men have an 18% lower cone a-wave amplitude, 23% lower cone b-wave amplitude, 24% lower rod b-wave amplitude, and a 25.5% lower mixed cone and rod b-wave amplitude.^78^ This sexual dimorphism does not only prevail in the healthy population, but may be of particular interest in PD as it is a disease that is slightly more frequent in men than in women.^3^ In healthy individuals, a study tracking ERG changes with age (via contact lens electrodes) noticed a considerable overlap in amplitudes for females and males at all ages. Nevertheless, mean age-adjusted rod b-wave amplitude was significantly higher for females (145 microvolts) than for males (119 μV), and mean age-adjusted cone b-wave amplitude was also higher in females (66 μV) than in males (58 μV).^37^ The cause of slightly smaller amplitudes in males compared to females is unknown. However, it is hypothesized to be caused by a greater axial length (distance between the anterior surface of the cornea and the fovea) in males. An earlier study in healthy subjects corrected for magnification of each eye by measuring its refraction and axial length in evaluations of disk width, neuroretinal rim, as well as the cup. They found that there was no statistical difference in the measurements between the sexes except for axial length, which confirmed that male eyes were slightly longer. The mean axial length of the male eyes was 23.96 ± 1.23 mm, and that of female eyes was 23.23 ± 1.24 mm.^79^ Previous reports have indicated that axial length, rather than refractive error, is a key factor contributing to reduced ERG response,^80,81^ and it is relevant in conditions like myopia.^82^ Altogether, this suggests a possible natural physiological restrain in aperture slightly impacting ERG readings so that females’ amplitude is naturally higher than that of males in ERG components.

Thus, ERG studies in PD cohorts should try to limit the interference of sex differences as they can impact their results’ statistical significance. Most studies have considered this when creating cohorts by including relatively equal proportions of both sexes. However, some have a substantially higher quantity of one over the other,^8,13,47^ and more importantly, rarely were sex-specific ERG anomalies considered. This raises the possibility that in the past, findings in female-dominated studies may not have been present in male-dominated ones. Even further, sex differences themselves may level out and result in no group findings, thus creating apparent contradictions in the search for a PD-specific ERG signature. We therefore propose that sex influences be considered essential as it pertains to natural ERG amplitude differences, but more specifically, as it is evident in disease incidence and development.^83–85^ As an essential factor, it would follow that all cohorts have a substantial sample size to accurately evaluate sex differences among participants. Lastly, when analyzing results, researchers should account for the sex-dependent reduction in amplitudes, especially when distinguishing PD anomalies from cohort-specific characteristics.

### Disease severity and duration

The third factor of importance is the correlation of the ERG readings with disease severity and duration. In PD studies, it is a regular practice to construct a cohort with a tight disease stage range or one that tracks the evolution of the disease. However, less often do studies also constrict this gap to disease duration. Multifocal ERG studies, examining cone central retinal function, revealed a generalized decrease in retinal electrical activity with a significant correlation between the functional changes in the retina (root-mean-square signal and P1 wave amplitude) and both disease duration and severity.^86^ This disease severity and duration effect has also been observed in similar tests. Pattern-ERG studies have shown a specific deficit at medium spatial frequency, particularly at a critical spatial frequency of 4.6 cycles per degree. Consequently, the reduced medium-to-low spatial frequency, termed ‘Pattern-ERG tuning ratio’, worsens with the clinical stage but subsequently improves with levodopa administration in PD cases comprised of stage II and III patients. The depression in amplitude seen became lost in cohorts also comprising stage I individuals thus highlighting the strong correlation with disease severity.^55^ In visual evoked potentials studies, there has also been a reported decline of amplitude and latency correlated with disease duration^44^ and a prolonged latency delay with motor impairment to disease stage.^43^ As the above-mentioned electrophysiological tests are often used alongside full-field ERG, the changes seen with disease severity and duration here are expected to produce a similar impact, especially when observed as bivariate correlations.^8^ Therefore, these factors should not be overlooked when constructing cohorts with wide ranges of disease severity and duration. In the past, PD studies have utilized patients with Hoehn-Yahr scores ranging from stages 1 to 3^29^ representing a broader range than those focused on scores of 1.1 ± 0.2^30^ or 2.14 ± 0.96.^31^ It can be considered that variability in data reported thus far may be due to ERG components identified at different points of disease progression, and thus not comparable. In other words, considering ERG signatures to represent PD without regard to which stage is in question becomes a plausible reason for the lack of reproducibility. We, therefore, propose that studies keep this in mind not only when aligning a cohort layout with a study's aim, but more importantly, when comparing results among cohorts that are otherwise not similar.

### Medication intake or administration

The fourth factor to consider with ERG testing on PD patients is medication intake. Depending on the stage severity, patients may or may not be regularly administrated PD medications, which can affect readings. In a study investigating the effects of levodopa administration, recordings were taken 1 h after intake. The tests lasted around 90 min, which included the period of peak levodopa blood levels attained 1.2 ± 0.3 h after a single oral dose. While no significant changes were reached, there was a clear tendency for an increase in b-wave amplitude with levodopa administration in the cone-dominated ERG, mixed rod and cone ERG, and steady-state flicker ERG. This effect is supported by additional testing that focused on the administration of other drugs such as antidopaminergic haloperidol. This agent was associated with a significant b-wave delay in the mixed rod and cone ERGs, cone-dominated ERGs, and steady-state flicker ERGs, with the most consistent implicit time delays being related to the steady-state flicker ERGs. In other words, antidopaminergic agents significantly delay ERG b-wave implicit times and oscillatory potentials, while levodopa tends to increase ERG amplitudes in healthy subjects.^87^ In the past, ERG studies performed on PD cohorts have shown that levodopa infusions ameliorate decreased parameters in patients. The reduction seen in the rod b-wave amplitude of the scotopic test was ameliorated with levodopa infusions by 12% across all intensities, by 12% in photopic a-wave amplitudes, and by 24% in photopic b-wave amplitudes. Prolonged b-wave latency in the photopic response was similarly ameliorated by 2%.^29^ This has also been the case for pattern-ERG studies, where amplitude was improved,^49,55^ and in visual evoked potentials and pattern-ERG studies, where latency for both waves was reestablished^48^ with the infusion of levodopa. It should be noted that there are also contradicting reports that hinder a consensus from being reached,^8,47^ however, from this, it can be concluded that the systemic administration of drugs can result in some general pharmacologically-induced changes in ERG patterns.

Depending on the aim of each team's research, we recommend first considering whether it is ideal and feasible to include the participants’ regular medication intake or not. The window of peak blood levels of a drug can be avoided easily but must be consistently applied. The latter avenue is not without substantial ramifications as teams need to not only consider the length of the withdrawal period, but also the time-dependent deterioration participants will undergo. Should the ERG be proposed as a biomarker in PD, it would be rendered sub-optimal if its success rate depended on patients being willing to forfeit the source of their symptom alleviation. Moreover, even when past studies took this into consideration,^31^ we cannot overlook that systematic alterations may already be produced with continuous administration. We, therefore, propose that to sediment the ERG as a biomarker of disease progression, a robust study would comprise two phases following participants with and without the influence of Parkinsonian medication. The aim would be to find ERG components that remain unaffected by medication and thus build ERG signatures that have diagnostic potential without the need to withhold medication. Acknowledging there is also a substantially greater variety of non-parkinsonian medications that may impact ERG studies, we encourage researchers to account for them rather than exclude them from their cohorts. After all, the features of a solid biomarker would include a high sensitivity of detection that stems from the population afflicted by PD regardless of demographic and co-morbidity characteristics. The identification of the optimal disease signature could facilitate its application to suspected prodromal cases, thereby advancing the utility of a progression biomarker into an early diagnostic tool for PD.

### Psychiatric distinctions and co-morbidities

Lastly, in aiming to use the ERG to identify PD anomalies, we also cannot fail to consider that ERG is a tool that has been used as a potential biomarker in studies of bipolar disorder (BP), schizophrenia (SZ), major depressive disorder, and autism spectrum disorder. The limitations become clear in comparison, as all of these have more detailed protocols that provide reproducible and viable data. It would, therefore, be beneficial to optimize ERG protocols for PD in a similar matter. Not only to mirror optimal standards but perhaps, more importantly, to ensure that anomalies in PD studies can be differentiated from changes seen in other disorders with higher accuracy.

In the case of SZ, ERG studies have been able to account for the factors discussed with better efficacy. In an early case study, when compared to control subjects, SZ subjects had a significantly reduced cone a-wave amplitude independent of the dose of antipsychotic agents taken.^88^ Some years later, a study performed on high-risk offspring with one affected parent by DSM-IV SZ or BP, found an anomaly that included a significantly lowered rod ERG b-wave amplitude at maximal velocity that was not affected by sex, age, or season of testing and was independent of the parental diagnosis.^89^ More recently, a study of SZ and BP cohorts found a reduced cone a-wave amplitude, a prolonged b-wave latency, and reduced mixed rod-cone a- and b-wave amplitudes. However, from these, a reduced cone b-wave amplitude was present only in the SZ cohort. The ERG anomalies observed allowed for the construction of a model using multiple stepwise logistic regressions. The comparison of predicted group membership to the actual membership was used to derive estimates of the model's sensitivity and specificity where the established model was able to distinguish SZ from control cohorts with 0.91 accuracy, 77% sensitivity, and 91% specificity. The accuracy of distinction was also similar for the BP cohort with 0.89 accuracy, 76% sensitivity, and 88% specificity. Furthermore, the SZ and BP cohorts could be differentiated with an accuracy of 0.86, 80% sensitivity, and 82% specificity.^65^ That is to say that the ERG anomalies observed allowed for specificities to be drawn for these and potentially other psychiatric disorders. As it is now, ERG studies that focus on SZ and BP have a much more stable ERG protocol and cohort criteria that allow them to find anomalies independent of factors such as medications, age, sex, and time of recording. Thus, allowing them to identify affected individuals from healthy individuals or patients with other conditions.

Similarly, the ERG has revealed certain abnormalities persistent among patients with major depressive disorder and autism spectrum disorder, both of which are much more closely associated with dopaminergic activity and parkinsonism.^90,91^ Taking into account medicated and drug-free cases, the electroretinographic signature for major depressive disorder cases center around an increased cone b-wave latency, reduced mixed rod/cone a- and b-wave amplitude, as well as a prolonged mixed rod/cone b-wave implicit time.^92^ Other teams have supported such findings (inclusive of serotonin norepinephrine reuptake inhibitor treatment response) by reporting a reduced rod b-wave amplitude.^93^ Autism spectrum disorder on the other hand, is a smaller field that has had to overcome the limitations of small cohort sizes.^94^ Originally reporting reduced rod b-wave amplitudes,^95^ teams have since replicated such discovery and additionally found reduced cone b-wave amplitudes and increased rod b-wave latencies.^96^ The latest studies added onto the ERG anomalies attributed to autism via decreased cone a- and b-wave amplitudes and increased cone b-wave latencies.^97^ Through multiple ERG protocol administration and tailoring, electroretinography work within major depressive disorder and autism spectrum disorder are a clear example of the importance of negative results^98,99^ and reproducibility^100^ on the path to establishing an ERG signature typical to a certain condition.

We thus continue to uphold that ERG studies in PD cases could improve by accounting for similar ERG factors as prior work done in alternative psychiatric conditions. We also propose that the avenue for PD biomarker research via the ERG can go further by taking into consideration the possibility of subjects with certain eye conditions and co-morbidities. Cataracts can reduce and delay ERG responses,^101^ but cataract removal surgery typically results in only a transient increase,^102^ with post-operative characterization often showing no significant difference.^103^ Systemic metabolic disorders should also be considered, as they have previously been reflected in retinal physiology. Liver and kidney disease, and drugs that affect these organs, usually reduce ERG b-wave amplitudes, particularly the scotopic dim flash ERGs. Moreover, patients with a failing liver, reduced small bowel, or undergoing bariatric surgery for morbid obesity are susceptible to significant loss of night vision and acuity due to limited availability or absorption of Vitamin A.^104^ However, we equally propose that neurological and psychiatric conditions in particular hold great relevance within the manifestation of PD. Patients are often impacted by mood disorders like depression and anxiety.^105–107^ Not often do PD studies list psychiatric disorders as exclusion criteria, perhaps to remain representative of the condition. Nonetheless, perhaps a lighter measure can be put in place when constructing cohorts so that the presence of such co-morbidities can be viewed as covariates and the results can remain representative. Such approach would retain the sensitivity and specificity of the ERG, ensuring that the biomarker signature remains for the most part, PD-specific.

Altogether, the cohort criteria previously mentioned should be encompassed when designing experimental and control groups in ERG studies of PD patients. First, patients’ age ranges should preferably be designated so that a moderate aging effect is tolerated rather than a drastic deterioration. This would allow the ERG anomalies to account for the natural changes that result from aging. Second, sex-specific differences should be noted when constructing a cohort to either encompass a whole population or investigate changes specific to males or females. Third, not only disease severity but also disease duration should be more tightly regulated as these can impact recordings. Fourth, medication intake should be considered when analyzing results. PD medications, as well as secondary medications, can have diverse effects on ERG readings so results might reflect the effect of the drug rather than that of the disease. As such, we recommend that cohorts be constructed within a tightly regulated age group with similar disease stages and duration so that the effect of these factors can approach a negligible point in the results produced. Furthermore, we acknowledge that a medication withdrawal period would help in isolating the ERG signature in PD without interference from drug effects, but ultimately, we suggest that cohorts aiming to reflect a whole population would provide a more accurate depiction. In perspective, once a PD-specific ERG signature has been identified and established, further testing would be required to validate its use in less controlled settings that would otherwise be more inclusive of a patient's living conditions. Leveraging recent technological advances, waveform data could then be deconstructed^108^ and analyzed through machine learning,^109–112^ further enhancing its diagnostic value and potentially improving its applicability in real-world clinical environments.

## Conclusions

When seeking to use full-field ERG anomalies as a possible biomarker in PD, we must note the great potential it has, given that this technique has been used with other disorders like SZ, BP, major depressive disorder, and autism spectrum disorder. However, we propose that what keeps us from using ERG in the field of PD with the same level of progress or success is a lack of consistency in studies that report different results. To this end, we recommend that the ERG and cohort factors that have been herein discussed be more closely observed. We should be aware of the effects that individual adjustments can have on the validity and reproducibility of the data. By minimizing the interference of these factors, as well as any interference from eye pathologies, ERG studies will be able to produce data that more accurately depicts the anomalies unique to PD. Once this is accomplished, we should then consider how we can maintain the same pattern while being more comprehensive of the population affected by PD. Should we be able to isolate unique PD anomalies, how would we use them to differentiate between conditions like major depressive disorder, SZ, BP, and metabolic disorders, which may have overlapping anomalies? How would we be able to adjust exclusion criteria to broaden the population studied while maintaining the signature already identified? If our ultimate goal is to use the full-field ERG as a potential biomarker of PD, these are questions that sooner or later have to be addressed. We propose that while we look to these other studies for guidance on improving PD-specific ERG protocols, we aim to optimize and amend our initiatives in the field. This would be the first progressive step to validating the use of the ERG as a potential early biomarker for PD, equipping us with a powerful non-invasive aid to diagnosis that we otherwise do not possess.
